# First manifestation of multiple sclerosis after immunization with the Pfizer-BioNTech COVID-19 vaccine

**DOI:** 10.1007/s00415-021-10648-w

**Published:** 2021-06-11

**Authors:** Joachim Havla, Yannick Schultz, Hanna Zimmermann, Reinhard Hohlfeld, Adrian Danek, Tania Kümpfel

**Affiliations:** 1 Institute of Clinical Neuroimmunology, LMU Hospital, Ludwig-Maximilians-Universität München, Munich, Germany; 2 Data Integration for Future Medicine (DIFUTURE) Consortium, Ludwig-Maximilians-Universität München, Munich, Germany; 3 Department of Neurology, LMU Hospital, Ludwig-Maximilians-Universität München, Munich, Germany; 4 Institute of Neuroradiology, LMU Hospital, Ludwig-Maximilians-Universität München, Munich, Germany; 5 Munich Cluster for Systems Neurology (SyNergy), LMU Hospital, Ludwig-Maximilians-Universität München, Munich, Germany

Dear Sirs,

Vaccination against SARS-CoV-2 is critical to control the pandemic. Although there are not yet sufficient data regarding the COVID-19 risk of patients with multiple sclerosis (MS), it is likely that especially older MS patients with higher levels of disability and relevant comorbidities have a higher risk of complications from COVID-19 infection [1]. Therefore, vaccination against the SARS-CoV-2 virus is generally recommended in MS, as is vaccination against other infectious agents [2]. It is thought that vaccine-induced protection from infection by far outweighs the risk of autoimmune exacerbation. Regarding vaccination against SARS-CoV-2 virus, three cases of reactivation or new-onset demyelinating disease were reported after vaccination with Oxford-AstraZeneca COVID-19 recombinant adenovirus (ChAdOx1 nCoV-19; AstraZeneca) [3]. Professional societies and physicians are currently addressing vaccination concerns with an awareness campaign to promote high vaccination rates in the MS community who generally tends to be more skeptical about vaccination [4]. The vaccination campaign is supported by initial safety data in MS: a very recently published study in approximately 500 MS patients showed that the relapse rate after vaccination with the Pfizer-BioNTech COVID-19 vaccine was similar (approximately 2%) to the relapse rate in a comparative time period without vaccination [5]. Here we report on a vaccinated patient who experienced the initial clinical manifestation of MS on a background of previously unknown, but likely pre-existing subclinical inflammatory CNS disease.

A 28-year-old woman developed the first clinical manifestation of relapsing MS after vaccination with the Pfizer-BioNTech COVID-19 vaccine (BNT162b2, Comirnaty©, BioNTech/Pfizer). Six days after the initially well-tolerated first immunization, she began to develop left abdominal neuropathic pain, sensory impairment below the T6 level, with hypoesthesia of right abdominal wall and genital regions, and left leg paresis. Magnetic resonance imaging (MRI) of the spinal cord on day 18 after vaccination showed a contrast-enhancing lesion at the T6 level, suggestive of myelitis, and cerebral MRI revealed multiple (> 20), partially confluent lesions with spatial dissemination but no Gadolinium enhancement. On cerebrospinal fluid (CSF) analysis mild pleocytosis (7 cells/µl) and oligoclonal bands were found. In line with a positive vaccine reaction, SARS-CoV-2 S antibodies (abs, IgG; Roche) were detected in serum (50.8 U/ml, 37 days after vaccination). SARS-CoV-2 infection was excluded on the basis of a negative PCR and absence of antibodies against the SARS-CoV-2 N protein (abs; Roche). The patient´s history was unremarkable with respect to previous relapses. Family history was positive for MS in a paternal cousin. After relevant differential diagnoses were excluded we diagnosed relapsing MS according to the 2017 McDonald criteria and initiated high-dose glucocorticoid therapy (1000 mg methylprednisolone i.v. for five days). Because complete remission of symptoms did not occur even after a second cycle of glucocorticoid therapy (2000 mg methylprednisolone i.v. for five days), we are currently escalating the relapse therapy with plasma exchange treatment, which resulted in further improvement to date (Figs. 1, 2).

**Fig. 1:**
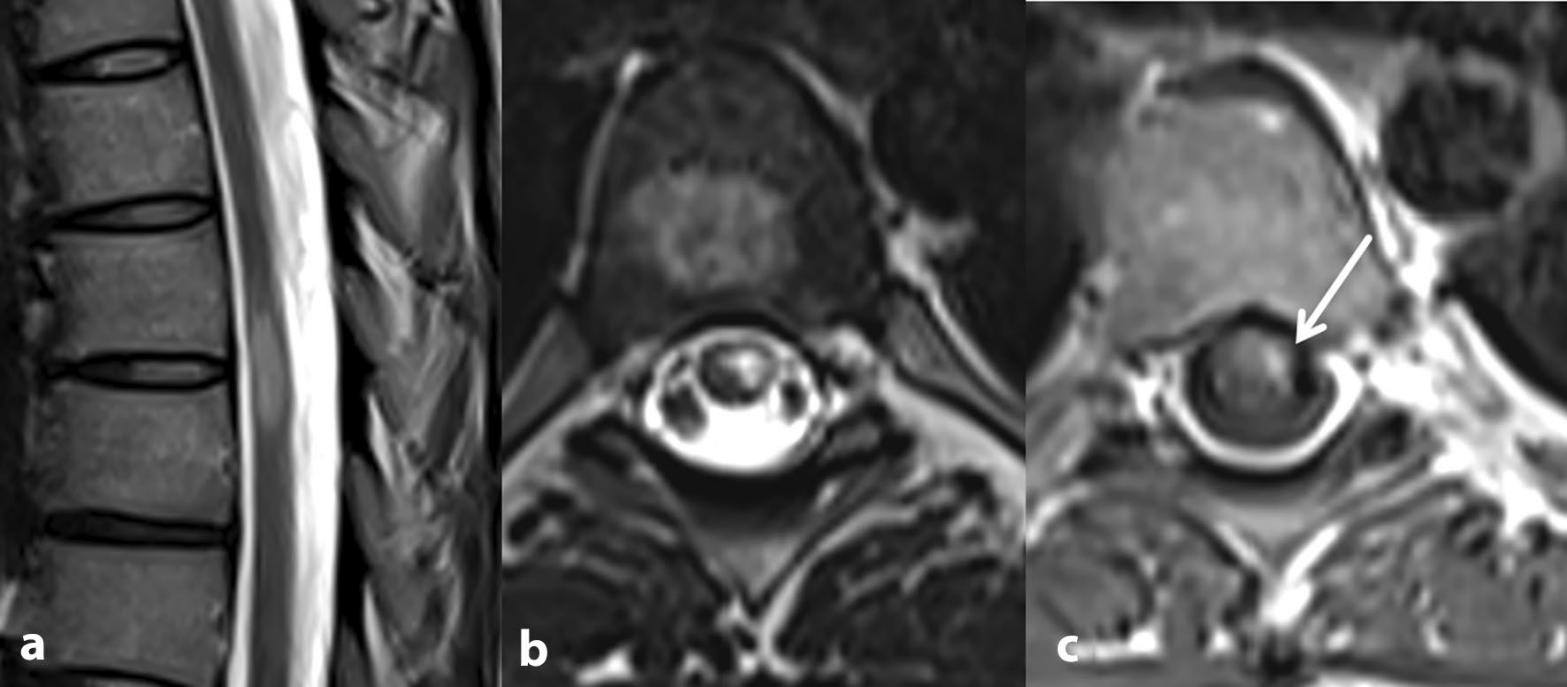
Spinal MRI: **a**, **b** peripherally located, T2 hyperintense lesion at level T6 and T7. The craniocaudal extension is less than two vertebral body segments. **c** Contrast enhancement after application of gadolinium is consistent with an active lesion. Thus, the criteria of spatial dissemination are fullfilled

**Fig. 2:**
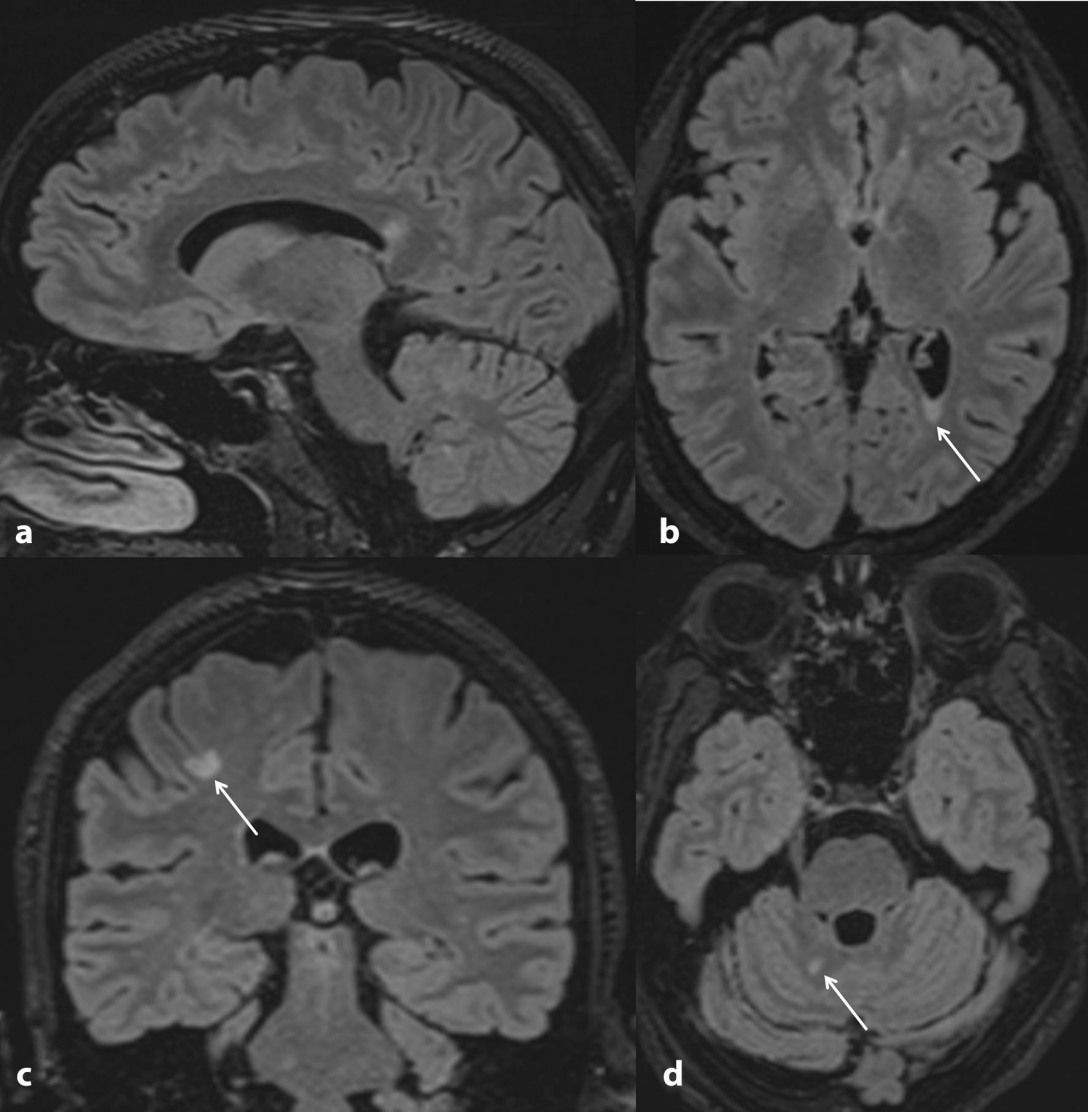
Cranial MRI performed one week after spinal MRI (Fig. 1). 3D FLAIR with 1 mm slice thickness and reconstruction in three planes. **a** The sagittal image shows a lesion in the splenium of the corpus callosum. **b** Axial image shows a periventricular lesion with triangular configuration. **c** Coronal image depicts a juxtacortical lesion involving the U-fibers. **d** Axial image shows involvement of the cerebellum. Overall, the MRI showed more than 20 specific lesions larger than 3 mm at periventricular, cortical/juxtacortical, or infratentorial locations without contrast enhancement

We are not aware of any published cases of initial MS manifestation after vaccination with Pfizer-BioNTech COVID-19 vaccine. Based on an individual case it is impossible to decide whether this occurrence is causally linked to vaccination or a mere coincidence. The Paul Ehrlich Institute (PEI, Federal Institute for Vaccines and Biomedicine), the German authority for vaccine safety monitoring, mentions three cases of myelitis after SARS-CoV-2 vaccination in its regularly updated database (last summary dated April 30^th^, 2021). One of these occurred after vaccination with the Pfizer-BioNTech COVID-19 vaccine (https://www.pei.de/EN/newsroom/dossier/coronavirus/coronavirus-content.html;jsessionid=2F08C732D73D6104723D32D08DD47942.intranet221?cms_pos=5; 10.05.2021).

Currently, the European Medicines Agency (EMA) has approved several vaccines to address the SARS-CoV-2 pandemic and additional vaccines are under regulatory review [3, 6, 7]. Assuming that some of these vaccines do carry a small risk of autoimmune exacerbation, it is still unclear whether and how this might differ between the different vaccines and whether patients with pre-existing inflammatory CNS disease should be prioritized for any particular vaccine. On the other hand, large population-based cohort analyses have shown that vaccine-preventable infections can trigger relapses and contribute to disease progression in patients with MS [8]. Consistent with this, individual case reports and a very recent cohort study suggest that also COVID-19 disease may be associated with an increased risk of relapse [9, 10].

Weighing these different risks, the infection-associated risks appears to be far greater than the risk of (re-)activation of MS disease activity associated with SARS-CoV-2 vaccination. Therefore, it is strongly recommended that all MS patients should be vaccinated against SARS-CoV-2.

It remains to be noted that the safety and efficacy of the SARS-CoV-2 vaccination campaign for MS patients needs to be supported by study data in the near future. For now, the rarity of case reports such as ours (compared with approximately 1.37 billion (10^9^) doses of vaccine administered worldwide, equivalent to 18 doses per 100 persons, as of May 14, 2021; https://www.nytimes.com/interactive/2021/world/covid-vaccinations-tracker.html) supports the view that the benefits of vaccination against SARS-CoV-2 far outweigh the potential risks.

## Declarations

### Availability of data and material (data transparency)

The datasets generated during and/or analyzed during the current study are available from the corresponding author on reasonable request.
